# Editorial: Modulating Prokaryotic Lifestyle by DNA-Binding Proteins: Learning from (Apparently) Simple Systems

**DOI:** 10.3389/fmolb.2016.00086

**Published:** 2017-01-06

**Authors:** Tatiana Venkova, Antonio Juárez, Manuel Espinosa

**Affiliations:** ^1^University of Texas Medical Branch (UTMB)Galveston, TX, USA; ^2^Departamento de Genética, Microbiología y Estadística, Universidad de BarcelonaBarcelona, Spain; ^3^Institut de Bioenginyeria de Catalunya (IBEC)Barcelona, Spain; ^4^Centro de Investigaciones Biológicas (CSIC)Madrid, Spain

**Keywords:** DNA–protein interactions, gene regulation in prokaryotes, replication control, regulation of bacterial gene expression, global regulatory networks

Within the research in Molecular Biology, one important field along the years has been the analyses on how prokaryotes regulate the expression of their genes and what the consequences of these activities are. Prokaryotes have attracted the interests of researchers not only because the processes taking place in their world are important to cells, but also because many of the effects often can be readily measured, both at the single cell level and in large populations. Contributing to the interest of the present topic is the fact that modulation of gene activity involves the sensing of intra- and inter-cellular conditions, DNA binding and DNA dynamics, and interaction with the replication/transcription machinery of the cell. All of these processes are fundamental to the operation of a biological entity and they condition its lifestyle. Further, the discoveries achieved in the bacterial world have been of ample use in eukaryotes. In addition to the fundamental interest of understanding modulation of prokaryotic lifestyle by DNA-binding proteins, there is an added interest from the healthcare point of view. As it is well-known the antibiotic-resistance strains of pathogenic bacteria are a major world problem, so that there is an urgent need of innovative approaches to tackle it. Human and animal infectious diseases impose staggering costs worldwide in terms of loss of human life and livestock, diminished productivity, and the heavy economic burden of disease. The global dimension of international trade, personal travel, and population migration expands at an ever-accelerating rate. This increasing mobility results in broader and quicker dissemination of bacterial pathogens and in rapid spread of antibiotic resistance. The majority of the newly acquired resistances are horizontally spread among bacteria of the same or different species by processes of lateral (horizontal) gene transfer, so that discovery of new antibiotics is not the definitive solution to fighting infectious diseases. There is an absolute need of finding novel alternatives to the “classical” approach to treat infections by bacterial pathogens, and these new ways must include the exploration and introduction of novel antibacterials, the development of alternative strategies, and the finding of novel bacterial targets. However, all these approaches will result in a stalemate if we, researchers, are not able to achieve a better understanding of the mechanistic processes underlying bacterial gene expression. It is, then, imperative to continue gaining insight into the basic mechanisms by which bacterial cells regulate the expression of their genes. That is why our Research Topic hosted by Frontiers in Molecular Biosciences was timely, and the output of it offers novel and up-to-date points of view to the “simple” bacterial world.

## The Research Topic

From the beginning of announcing the present Topic, we stated that “our goal to achieve with this Topic of Frontiers was to accelerate and to broaden our understanding of gene regulation in prokaryotes through knowledge on protein–DNA interactions.” Thus, it was our aim that the topic would provide us with the opportunity of bringing together a number of very active researchers that were dealing with DNA replication, genetic regulation, as well as the approaches taken by the various proteins that play a role in these processes and their consequences. The papers compiled in this Research Topic will allow us to gain an up-to-date knowledge of some of the mechanisms of genetic regulation, DNA transfer, and DNA replication as well. Further, the readers will understand the influence of the topic on the scientific world, and will learn the most contemporary reasoning, results, and experimental approaches in the area. The authors of the articles which compose the Research Topic represent some of the top laboratories in the world actively working on the Topic of DNA-binding proteins from prokaryotic origin. We are aware that the list of contributors is far from complete. But logical limitations must be introduced. The present Research Topic brings together important articles dealing with the study of gene regulation and the mechanisms underlying, including replication, transfer, segregation, and transcriptional and post-transcriptional control of gene expression as well as global regulatory networks in prokaryotes. Finally, we envisage that a large number of people will notice the topic subject and will thereby come to realize that it is of great importance, broadening the expected impact. Hence, it seems important to reunite, in a single and intense Research Topic the articles written by researchers that give the latest information from the various fields of structural analysis with those who provide genetic, biochemical, and biophysical information.

We have gathered a total of 24 articles written by a total of 114 authors, thus we believe the joint contributions has reached a high level of contributors that have achieved a high scientific standard that will be difficult to overcome in future attempts in this particular field. We are pleased to present articles covering not only basic research, but also applied science. The readers will be able to look at the bacterial replication control in terms of time (Riber et al.), mechanistic concepts (Wegrzyn et al.), novel ways to achieve DNA replication (Salas et al.), basic principles of DNA strands' opening (Jha et al.), and a novel regulation of a replication initiator protein by inorganic compounds (Ruiz-Masó et al.). Segregation and partition of DNA copies to daughter cells (Oliva; Funnell et al.) will help the readers to understand the basis of this fundamental process: how cells receive copies of DNA lies at the basis of bacterial life. Control of bacterial gene expression has been tackled at different levels. Firstly, gene-expression control at the level of transcription covers various aspects including RNA Polymerase interactions (Lee and Borukhov), environmental signal sensing (Hartman et al.), and genome-wide indirect regulation via protein–DNA interactions at a distance causing large-scale restructuring of the chromosome (Qian et al.). Secondly, global regulatory networks (Solano-Collado et al.; Erill et al.; Cech et al.; Brandi et al.) as well as post-transcriptional control (Amin et al.) that lead to intra-chromosomal communications and structural arrangement by DNA-interacting proteins have been a subject of very active research. And thirdly, the role of bacterial operons encoding toxin-antitoxin genes, and how they regulate their expression has been reviewed from the structural and functional points of view by Chan et al. Deeper understanding of general biological processes like replication and transcription in bacteria favors the quest for novel anti-bacterial agents, a life-saving area of research that is discussed by the Lee and Borukhov. The bacterial conjugation studied as a major pathway for horizontal gene transfer that leads to the spread of antimicrobial resistance, has been approached by Grohmann et al., Gruber et al., Fernández-González et al., and discussed in detail by Fernandez-Lopez et al. On the other hand, extensive research on human pathogens as *Salmonella* (Lobato-Márquez et al.), *Shigella* (Letzia Di Martino et al.), and Gram-positive bacteria (Albanesi and de Mendoza) provides not only better understanding of their molecular genetics, but also could become novel diagnostics, anti-virulence, and biotechnological tools. The relatively recently discovered bacterial immune system CRISPR, applied in eukaryotic genomic engineering and soon in human medicine, is also tackled in a theory article presenting a mathematical model on the role of plasmid/phage copy number in the process (Severinov et al.).

## Where do we go from here?

We have arrived in the post genomic era in a somewhat embarrassing position. We now know the genomic content of a large number of organisms and the gene expression patterns in many of them. We indeed are in the situation of trying to handle big data buried within the many little crevices and nooks of the Internet resources. However, for the minority of gene products whose cellular function is presently known, only occasionally we know how the protein product of a gene actually works or how it interacts with other components in the cell. This situation is equivalent to being able to understand Morse code, but not yet understanding the language of the person sending us the telegraphic message. We should aim to a comprehensive view of regulatory systems which for the most part are not yet fully understood. In addition, we should learn more from the mechanisms of action of some of proteins that play central roles in global regulation of cellular function. Prokaryotic systems need to be emphasized because in general these systems are considerably better understood than their eukaryotic counterparts are. This is because work with prokaryotes allows highly sophisticated genetic analyses, complex and sensitive physiological measurements, and detailed biochemical and biophysical studies of the components of regulatory systems. It is with prokaryotic systems that the goal, now distantly visible on the horizon, of achieving a basic understanding of all the biological processes within a cell and their interactions, will first be achieved. Novel search engines must be developed, so that we can use the many data that are nowadays at our disposal, but we must find ways and procedures on how to dig them out, how to order them and clarify their meaning and how to be able to develop models in which we can express or views of the prokaryotic world. Sharing this information among scientists that try to understand the complexity of the prokaryotic world should constitute the next generation of knowledge and toward the today science, in our opinion, should devote the next coming years.

## Author contributions

All authors listed, have made substantial, direct and intellectual contribution to the work, and approved it for publication.

## Funding

While this Editorial was written, authors were funded by Spanish MINECO, grants CSD2008-00013, BIO2013-49148-C2-2-R, BIO2013-49148-C2-1-R, BIO2015-69085-REDC, BIO2016-76412-C2-2-R-AEI/FEDER, UE, and BIO2016-76412-C2-1-R-AEI/FEDER, UE.

### Conflict of interest statement

The authors declare that the research was conducted in the absence of any commercial or financial relationships that could be construed as a potential conflict of interest.

